# Lichen Sclerosus Atrophicus [LSA] in the Areolae: A Case Report

**DOI:** 10.1155/2012/825963

**Published:** 2012-10-31

**Authors:** L. Padmavathy, L. Lakshmana Rao, M. Dhana Lakshmi, N. Sylvester, N. Ethirajan

**Affiliations:** ^1^Dermatology, Urban Health Centre, RMMC, Annamalai University, B3, RSA Complex, Annamalai Nagar 608002, India; ^2^Division of Pathology, RMMC, Annamalai University, Annamalai Nagar 608002 T.N., India; ^3^Division of Community Medicine, RMMC, Annamalai University, Annamalai Nagar 608002 T.N., India

## Abstract

Lichen sclerosus (LS) is a chronic inflammatory disorder of an unknown aetiology most commonly affecting the anogenital area. However, extragenital involvement also occurs uncommonly. A case of extra-genital LS involving the areolae of both breasts, in a 15-year-old boy, is reported for its rarity.

## 1. Introduction

Lichen sclerosus (LS) is a chronic inflammatory disorder of an unknown etiology. Anogenital areas are the most common sites of involvement, with or without extragenital involvement. Isolated extragenital involvement is rare and, more so, bilaterally symmetrical involvement of the areolae of both breasts. A case of LS affecting the areolae of breast in a 15 year-old boy is reported.

## 2. Case Report

**Figure 1:**
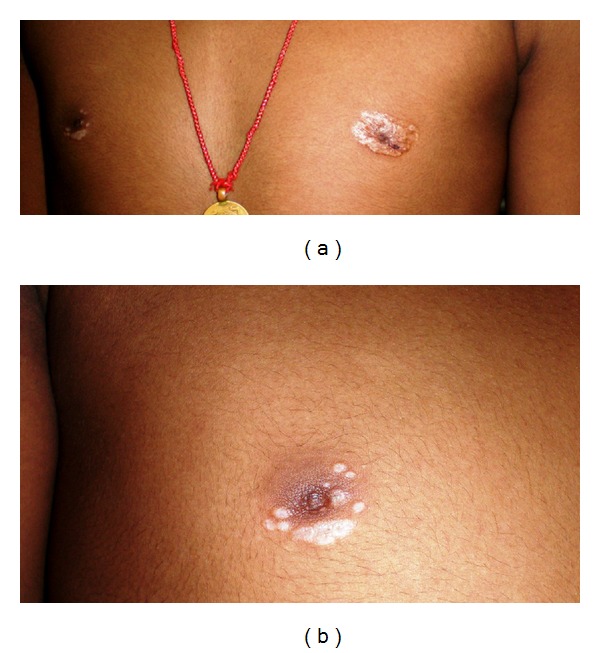
Hypopigmented and depigmented, polygonal atrophic plaques with “delling” about 3 × 3.5 cm on left and 2 cm × 1 cm on the right areola. Some papules coalesced to form plaques with comedo like plugs on the surface, more marked and larger on the left areola with minimal scaling over the plaque. A tiny hemorrhagic vesicle over the lesion on left side.

A 15 year-old boy presented with asymptomatic, hypopigmented and depigmented macules and plaques on the areolae of both breasts of 2 years, duration. On examination, hypopigmented and depigmented, polygonal atrophic plaques with “delling” about 3 × 3.5 cm on the left and 2 cm × 1 cm on the right areola were seen. Some papules coalesced to form plaques with comedo-like plugs on the surface, more marked and larger on the left areola. There was a minimal scaling on the plaque. A tiny hemorrhagic vesicle was seen on the lesion on the left side [Figure 1].

 There were no genital symptoms or lesions. Systemic examination did not reveal any abnormality. The routine and relevant biochemical investigations were noncontributory. LE cell test and ANA test were negative. While the biopsy was being attempted, the skin felt very fragile and the epidermis got detached very easily, even before the biopsy wound could be sutured. 

**Figure 2:**
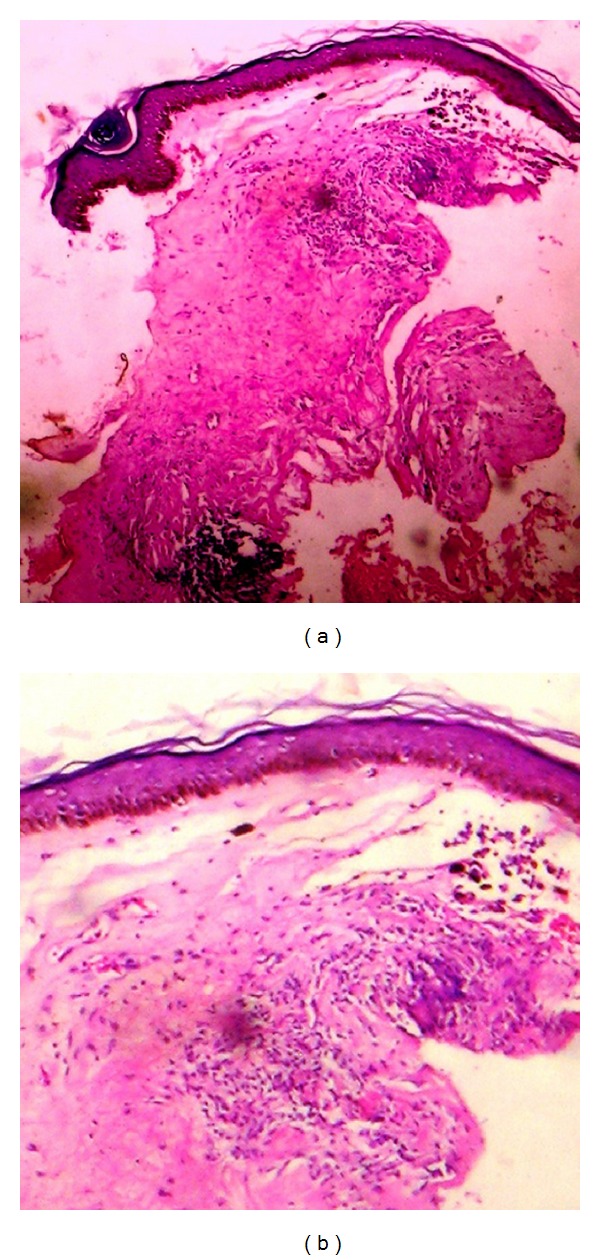
Hyperkeratotic scale with follicular plugging and atrophic epidermis. Sub-epidermal zone of pallor (edema) and scattered inflammatory cells.

 Histopathological examination of the plaque from the lesion on the left side revealed hyperkeratotic scale with follicular plugging and atrophic epidermis. There was a subepidermal zone of pallor (edema); and scattered inflammatory cells were present. The features were reported to be compatible with LS (Figure 2).

The patient was prescribed topical clobetasol propionate and was advised frequent followups. 

## 3. Discussion

Lichen sclerosus et atrophicus, described originally by Hallopeau, in 1887 [1], is an infrequent, benign, chronic, and inflammatory dermatosis affecting both the epidermis and the dermis [2]. Typical findings are white opalescent papules that may cluster and progressively result in parchment-like skin [1, 3]. Lichen sclerosus (LS) encompasses the disorders known as LSetA, Balanitis xerotica obliterans (LS of male genitalia glans and prepuce), and kraurosis vulvae (LS of labia majora, labia minora, perineum, and perianal region [4]).

 Lichen sclerosus is relatively uncommon in adult women, rare in men and girls, and extremely rare in boys though our patient was a 15-year-old boy. While genital LS is associated with severe pruritus and burning, extragenital LS is reported to be asymptomatic, as observed in the present case. This is similar to the study in a large series of 33 patients reported from Korea [5].

Lichen sclerosus most commonly affects anogenital region (85%–98%). Extra genital LS can be seen in 15%–20% of the cases [6]. Common extra genital sites of involvement are trunk, sites of pressure, upper back, wrists, buttocks, and thighs [7], while in our patient areolae of breasts were affected. Atypical locations would be the palmar and plantar regions, nipples, scalp, vaccination sites, and the face, when the differential diagnosis should be made with discoid lupus and sclerodermia circumscripta [1]. The disseminated form of LS is poorly described in the literature and occurs in 15% to 20% of the cases [1]. 

 The exact etiology of LS is unknown [1]. Autoimmune, genetic, infective, hormonal, and local factors have been implicated. Familial cases and a significant association with HLA class II antigen DQ7 have been demonstrated [8]. Though infective cause like the spirochete *Borrelia* species is implicated, there are conflicting reports about its etiological role in studies from various authors [1, 4]. Local factors like friction, trauma, or rubbing may cause Koebner's phenomenon triggering LS [9]. This could be presumed to be a factor for the localisation of the lesions on the areolae, as the boy might be holding his books, school bag, and so forth, close to his chest, leading to friction and trauma.

 According to the literature, there is a 21.5% to 34% rate of association between this entity and autoimmune diseases, and 79% of cases had autoantibodies [3]. However, due to lack of facilities, immunological studies could not be undertaken, though ANA test and LE cell test were negative in our patient. Many biochemical abnormalities like alterations in distribution of tenascin, fibronectin, and fibrinogen in vulval lichen sclerosus are reported at a molecular level [10]. But the above investigations were not undertaken due to a lack of facilities and, more so, as our patient did not have any genital involvement.

Histopathologic findings of the extragenital LS shows more significant epidermal thinning and cleft formation compared to genital lichen sclerosus et atrophicus, which suggests that extra genital LS shows more evolved lesions [5]. Comedo-like plugs on the surface of the plaque correspond to dilated appendageal ostia corroborated by the histology of follicular plugging. The easy detachment of the epidermis when biopsy was being attempted could be presumed to be due to the flattened interface of the epidermis and dermis resulting in the fragility of the dermal-epidermal junction [11]. 

The various therapeutic modalities found effective in LS are potent topical glucocorticoid preparations (clobetasol propionate) for 6–8 weeks and intralesional corticosteroids. Topical calcineurin inhibitors like pimecrolimus and tacrolimus, topical vitamin A derivatives, and topical androgens were also tried with varying results [11]. Phototherapy with bath or cream PUVA therapy or UVA1 therapy was found beneficial.

Since the lesions were very well localised, the patient was prescribed only potent topical corticosteroid applications with an advice for frequent reviews.
